# Hepatocyte-Specific Deletion of AMPKα1 Results in Worse Outcomes in Mice Subjected to Sepsis in a Sex-Specific Manner

**DOI:** 10.3389/fimmu.2020.00210

**Published:** 2020-02-13

**Authors:** Satoshi Kikuchi, Giovanna Piraino, Michael O'Connor, Vivian Wolfe, Kiana Ridings, Patrick Lahni, Basilia Zingarelli

**Affiliations:** ^1^Department of Emergency Medicine, Ehime University, Toon, Japan; ^2^Division of Critical Care Medicine, Cincinnati Children's Hospital Medical Center, Cincinnati, OH, United States; ^3^Department of Pediatrics, College of Medicine, University of Cincinnati, Cincinnati, OH, United States

**Keywords:** AMPKα1, Cre-lox, cecal ligation and puncture, mitochondria, lung injury, liver injury, female sex

## Abstract

Alterations in the energy homeostasis contribute to sepsis-mediated multiple organ failure. The liver plays a central role in metabolism and participates to the innate immune and inflammatory responses of sepsis. Several clinical and experimental studies have suggested that females are less susceptible to the adverse outcome of sepsis. However, underlying mechanisms of organ damage in sepsis remain largely undefined. AMP-activated protein kinase (AMPK) is an important regulator of mitochondrial quality control. The AMPK catalytic α1 isoform is abundantly expressed in the liver. Here, we determined the role of hepatocyte AMPKα1 in sepsis by using hepatocyte-specific AMPKα1 knockout mice (H-AMPKα1 KO) generated with Cre-recombinase expression under the control of the albumin promoter. Using a clinically relevant model of polymicrobial sepsis by cecal ligation and puncture (CLP), we observed that male H-AMPKα1 KO mice had higher plasma levels of tumor necrosis factor-α and interleukin-6 and exhibited a more severe liver and lung injury than male H-AMPKα1 WT mice, as evaluated by histology and neutrophil infiltration at 18 h after CLP. Plasma levels of interleukin-10 and the keratinocyte-derived chemokine were similarly elevated in both KO and WT male mice. At transmission electron microscopy analysis, male H-AMPKα1 KO mice exhibited higher liver mitochondrial damage, which was associated with a significant decrease in liver ATP levels when compared to WT mice at 18 h after sepsis. Mortality rate was significantly higher in the male H-AMPKα1 KO group (91%) when compared to WT mice (60%) at 7 days after CLP. Female H-AMPKα1 WT mice exhibited a similar degree of histological liver and lung injury, but significantly milder liver mitochondrial damage and higher autophagy when compared to male WT mice after CLP. Interestingly, H-AMPKα1 KO female mice had lower organ neutrophil infiltration, lower liver mitochondrial damage and lower levels of cytokines than WT female mice. There was no significant difference in survival rate between WT and KO mice in the female group. In conclusion, our study demonstrates that AMPKα1 is a crucial hepatoprotective enzyme during sepsis. Furthermore, our results suggest that AMPK-dependent liver metabolic functions may influence the susceptibility to multiple organ injury in a sex-dependent manner.

## Introduction

Sepsis is a life-threating organ dysfunction caused by dysregulated host responses to infection (1). Sepsis is the most common cause of patient mortality in intensive care units, with a global incidence of ~18 million cases per year and a mortality rate of 28–40% (2). Although co-morbidities contribute to the clinical variability, numerous experimental and clinical studies indicate that sex-specific differences influence the susceptibility to sepsis and the subsequent multiple organ dysfunction syndrome (MODS) and mortality (3–5).

The liver plays a central role in metabolic and immunological homeostasis (6). Clinical studies have shown that liver dysfunction and failure are serious complication in sepsis and directly contributes to disease progression and death (6, 7).

Mitochondrial dysfunction has been proposed as an important cause of sepsis-related organ failure in sepsis (8, 9). AMP-activated protein kinase (AMPK) is a crucial sensor of energy status and contributes to several metabolic processes for energy homeostasis. This kinase consists of a catalytic α-subunit (α1 and α2) and two β and γ regulating subunits, which are allosterically activated by low levels of adenosine triphosphate (ATP) and high levels of adenosine monophosphate (AMP) (10–12). A key component of the metabolic effects of AMPK is the activation of mitochondrial biogenesis leading to improved cellular energy utilization (10). AMPK also contributes to activation of autophagy, a highly conserved catabolic process that degrades and recycles dysfunctional cytoplasmic constituents, including damaged mitochondria, thus ensuring a proper process of mitochondrial turnover (10, 13).

We have recently demonstrated that specific pharmacological activators of AMPK exert beneficial effects in sepsis and reduce hepatic, cardiac and pulmonary injury in experimental models using male mice (14–16). Specifically, in the liver, these beneficial effects are associated with amelioration of mitochondrial biogenesis and function (14).

Given the potential benefit for AMPK activators to attenuate sepsis-induced liver injury, further investigation is merited to determine the biologic role of AMPK within the liver. As AMPKα1 is abundantly expressed in the liver of rodents and the predominant isoform in human hepatocytes (11, 12, 17), we sought to investigate the role of the hepatocyte AMPKα1 on sepsis-induced liver injury and mortality by employing hepatocyte-specific AMPKα1 knockout (H-AMPKα1 KO) young male mice (18). In order to characterize the sexual dimorphism of liver injury, we also used young H-AMPKα1 KO female mice. In these loss-of function studies, we demonstrated that specific AMPKα1 gene deletion in hepatocytes was associated with increased susceptibility to sepsis-induced liver and lung injury and increased mortality in male mice only. On the contrary, hepatocyte-specific AMPKα1 deletion in female mice had a surprising protective effect in liver mitochondrial structure, lung injury and systemic inflammatory response. Thus, our data suggest that hepatocyte AMPKα1 is an important modulator of the metabolic response in sepsis; however, its function is sex-dependent.

## Materials and Methods

### Murine Model of Polymicrobial Sepsis

The investigation conformed to the National Institutes of Health Guide for the Care and Use of Laboratory Animals (Eighth Edition, 2011) and was approved by the Institutional Animal Care and Use Committee of the Cincinnati Children's Hospital Medical Center. Specific deletion of AMPKα1 in hepatocyte was achieved by using Cre-lox technology. Mice expressing Cre recombinase under the control of the albumin promoter [B6.Cg-Tg(Alb-cre)21Mgn/J] and AMPKα1flox/flox mice (Prkaa1tm1.1Sjm/J), both on C57BL/6 genetic background, were obtained from Jackson Laboratories (Bar Harbor, Maine) and were crossed to generate hepatocyte-specific AMPKα1 (H-AMPKα1) KO mice. Mice were housed in pathogen-free conditions under a 10 h light/14 h dark cycle with free access to food and water. Both male and female mice were used at 8–12 weeks of age. Mice were anesthetized with 2.0% isoflurane in 50% oxygen and polymicrobial sepsis was induced by cecal ligation and puncture (CLP) (19). A midline laparotomy was performed. After opening the abdomen, the cecum was exteriorized, ligated and punctured twice with a 23-G needle. The cecum was then returned into the peritoneal cavity and the abdominal incision was closed. Mice were resuscitated subcutaneously with 35 ml/kg 5% dextrose solution immediately after and at 3 h after the surgical procedure. Control mice did not undergo any surgical procedure. Mice were then sacrificed at 18 h after CLP. Blood, liver, and lungs were collected for biochemical assays.

### Survival Study

In a separate study, another cohort of mice (*n* = 20–23) was subjected to a milder model of CLP by puncture with 25-G needle and was used for assessing survival rate. Mice received fluid resuscitation (35 ml/kg normal saline with 5% dextrose subcutaneously) immediately after, at 3 h and every 24 h after the CLP procedure up to 72 h. To minimize pain at the surgical incision site, lidocaine hydrochloride (1%, 4 mg/kg total dose) was applied locally immediately after the procedure and every 12 h up to 48 h. Survival was monitored for 7 days.

### Myeloperoxidase Activity

Myeloperoxidase (MPO) activity was measured as an indicator of neutrophil infiltration in lung and liver tissue (20). Tissues were homogenized in a solution containing 0.5% hexa-decyl-trimethyl-ammonium bromide dissolved in 10 mM potassium phosphate buffer (pH 7.0) and centrifuged for 30 min at 4,000 × g at 4°C. An aliquot of the supernatant was allowed to react with a solution of tetra-methyl-benzidine (1.6 mM) and hydrogen peroxide (0.1 mM). The rate of change in absorbance was measured by spectrophotometry at 650 nm. MPO activity was defined as the quantity of enzyme degrading 1 μmol of hydrogen peroxide/min at 37°C and expressed in units per 100 mg weight of tissue.

### Histopathologic Analysis

Frozen liver sections and paraffin-embedded lung sections were stained with hematoxylin and eosin, and evaluated by two independent observers blinded to the treatment groups. Liver injury was evaluated on the presence of necrosis, sinusoid congestion and infiltration of red blood and inflammatory cells. Lung injury was evaluated on the presence of alveolar capillary congestion, infiltration of red blood cells and inflammatory cells into the airspace, alveolar wall thickness, and hyaline membrane formation.

### Plasma Alanine Aminotransferase (ALT)

Plasma levels of ALT was evaluated as index of liver function by an enzymatic assay kit (Sekisui Diagnostics, Charlottetown, Canada) using the protocols recommended by the manufacturer.

### Measurement of ATP Levels

Mitochondrial function in the liver was assessed by measuring ATP levels. Homogenates were obtained from fresh livers and were deproteinized with perchloric acid using a Deproteinization Sample Preparation Kit (BioVision, San Francisco, CA). Liver ATP levels were measured using an ATP Fluorometric Assay Kit (BioVision, San Francisco, CA). ATP levels were expressed as nmol/g tissue weight.

### Transmission Electron Microscopy

Liver samples were fixed in 3% glutaraldehyde, postfixed in 1% osmium tetroxide in sodium phosphate buffer, and cut with ultramicrotome. Samples were stained with 2% uranyl acetate and lead citrate. The sections were viewed and photographed on Hitachi H-7650 transmission electron microscope at 120 kV. The total number of mitochondria and autophagosomes, and the presence of abnormal or enlarged mitochondria with loose matrix, fragmented cristae and membranes were determined in 9 consecutive cells in four different sections for each animal by using NIH ImageJ analysis (21).

### Cytosol Extraction and Western Blot Analysis

Livers were homogenized in a buffer containing 0.32 M sucrose, 10 mM Tris-HCl (pH 7.4), 1 mM EGTA, 2 mM EDTA, 5 mM NaN3, 10 mM β-mercaptoethanol, 20 μM leupeptin, 0.15 μM pepstatin A, 0.2 mM phenylmethanesulfonyl fluoride, 50 mM NaF, 1 mM sodium orthovanadate, and 0.4 nM microcystin. Samples were centrifuged at 1,000 × g for 10 min at 4°C and the supernatants collected as cytosol extracts. Cytosol content of the light-chain (LC)3B-I and LC3B-II was determined by immunoblot analyses. Extracts were boiled in equal volumes of NuPAGE® LDS Sample Buffer (4X) and 40 μg of protein loaded per lane on a 16% Tris-glycine gradient gel. Proteins were separated electrophoretically and transferred to nitrocellulose membranes. For immunoblotting, membranes were blocked with 5% non-fat dried milk in Tris-buffered saline (TBS) for 1 h and incubated with primary antibodies for 24 h. Membranes were washed in TBS with 0.1% Tween 20 and incubated with secondary peroxidase-conjugated antibody; the immunoreaction was visualized by chemiluminescence and x-ray. Membranes were also re-probed with primary antibody against GADPH to ensure equal loading for cytosol proteins. Densitometric analysis of blots was performed using Quantity One (Bio-Rad Laboratories, Des Plaines, IL, USA).

### Plasma Levels of Cytokines

Plasma levels of tumor necrosis factor-α (TNFα), interleukin-10 (IL-10), interleukin-6 (IL-6), and keratinocyte-derived chemokine (KC) were evaluated by a commercially available multiplex array system (Milliplex, Millipore Corporation, Billerica, MA) using the protocols recommended by the manufacturer.

### Materials

The primary antibodies directed at LC3B-I and LC3B-II were obtained from Cell Signaling (Beverly, MA); the primary antibody directed at GADPH was obtained from Abcam (Cambridge, MA); the secondary antibodies were obtained from Santa Cruz Biotechnology (Santa Cruz, CA). Unless otherwise stated, all other chemicals were obtained from Sigma-Aldrich (St. Louis, MO).

### Statistical Analysis

Statistical analysis was performed using SigmaPlot 13.0 (Systat Software, San Jose, CA, USA). Data in figures and text are expressed means ± SEM of n observations (*n* = 4–8 animals for each group). The results were examined by analysis of variance followed by the Student-Newman-Keuls's correction *post hoc t*-test. The Gehan-Breslow and log-rank tests were used to compare differences in survival rates (*n* = 20–23 animals for each group). A value of *P* < 0.05 was considered significant.

## Results

### Hepatocyte-Specific Deficiency of AMPKα1 Results in Liver Damage After Sepsis in a Sex-Independent Manner

**Figure 1:**
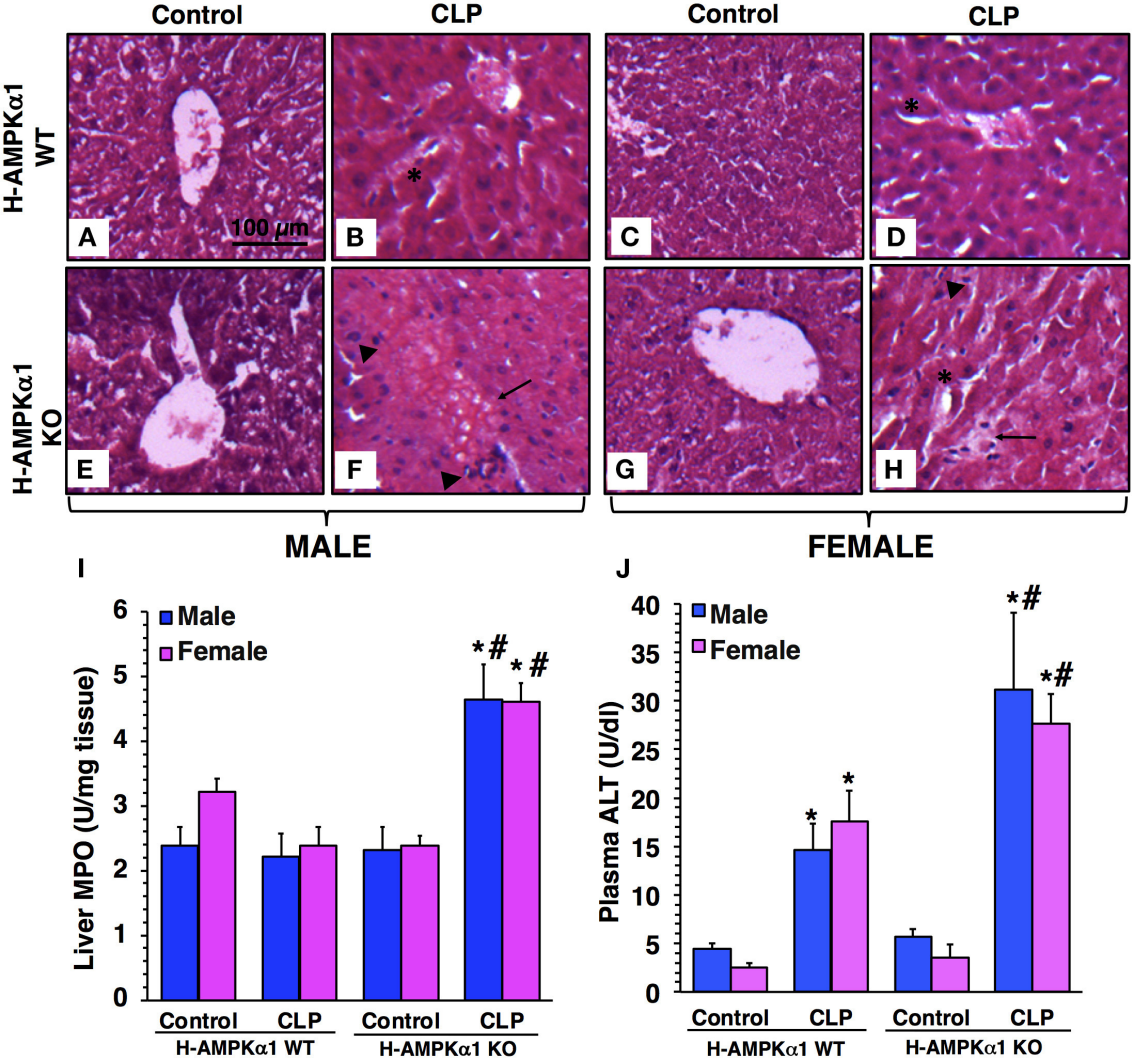
Representative histology photomicrographs of liver sections of hepatocyte-specific (H-AMPKα1) wild-type (WT) and knock-out (KO) mice at basal control condition and at 18 h after cecal ligation and puncture (CLP). Normal liver architecture in control male WT **(A)** and male KO **(E)**, and in control female WT **(C)** and female KO **(G)** mice. Liver damage in male **(B)** and female **(D)** H-AMPKα1 WT mice with modest areas of sinusoid congestion (asterisks). Liver damage in male **(F)** and female **(H)** H-AMPKα1 KO mice with necrosis (arrows) and infiltration of inflammatory cells (arrowheads). Magnification ×100; scale bar = 100 μm. A similar pattern was seen in *n* = 4–8 different tissue sections in each experimental group. Liver myeloperoxidase (MPO) activity **(I)**, plasma ALT levels **(J)** in male and female H-AMPKα1 WT and KO mice at 18 h after CLP. Data represents the mean ± SEM of 4–8 mice for each group. *P < 0.05 vs. sex-matched control mice; ^#^*P* < 0.05 vs. sex-matched WT mice.

At 18 h after CLP, both male and female WT mice exhibited liver damage with modest areas of necrosis and sinusoid congestion at histological examination. However, H-AMPKα1 KO mice of both sexes showed more prominent liver damage with significant necrosis, edema and infiltration of inflammatory cells (Figures 1A–H). To confirm the histological findings of liver neutrophil infiltration in KO mice after sepsis we measured the activity of MPO, a neutrophil lysosomal enzyme. At 18 h after CLP, there was no increase in liver MPO activity in male or female WT mice. However, both male and female KO mice exhibited a significant elevation of MPO after sepsis when compared to sex-matched WT septic mice (Figure 1I). To further quantify liver injury, we measured plasma levels of ALT. Both male and female WT mice exhibited a similar degree of plasma ALT levels at 18 h after CLP. However, male and female KO mice had significant higher levels of ALT after sepsis when compared to sex-matched WT septic mice, thus confirming a more severe liver injury in a sex-independent manner (Figure 1J).

### Hepatocyte-Specific Deficiency of AMPKα1 Results in Liver Mitochondrial Damage After Sepsis in a Sex-Dependent Manner

**Figure 2:**
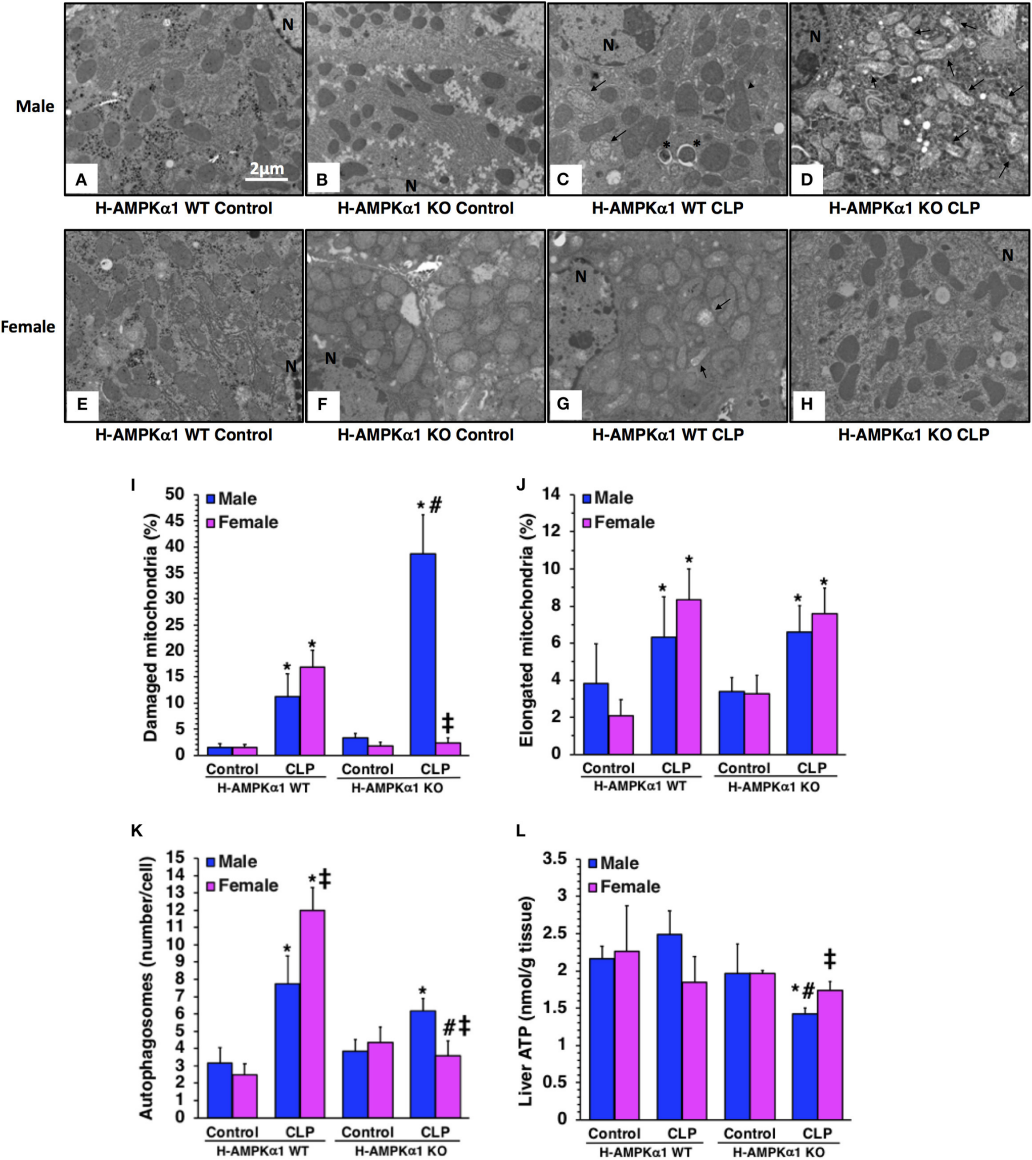
Transmission electron microscopy of hepatocytes in hepatocyte-specific (H-AMPKα1) wild-type (WT) and knock-out (KO) male **(A–D)** and female **(E–H)** mice at basal control condition or 18 h after cecal ligation and puncture (CLP). Arrows, damaged mitochondria presenting translucent matrix, disrupted membrane and cristae; arrow heads, elongated mitochondria; asterisk, autophagic vescicles packed with mitochondria; N, nucleus. Quantification of damaged mitochondria **(I)**, elongated mitochondria **(J)**, and autophagosomes **(K)** of hepatocytes of hepatocyte-specific (H- AMPKα1) wild-type (WT) and knock-out (KO) mice at 18 h after cecal ligation and puncture (CLP). Liver sections were examined at transmission electron microscopy. Damaged, elongated mitochondria and autophagosomes were determined by using the NIH Image J software and expressed as percentage of total number of mitochondria in nine consecutive cells. Data are means ± SEM of 3–4 mice for each group. Liver ATP content **(L)** of hepatocyte-specific (H-AMPKα1) wild-type (WT) and knock-out (KO) mice at 18 h after cecal ligation and puncture (CLP). Data represents the mean ± SEM of 4–6 mice for each group. *P < 0.05 vs. sex-matched control mice; ^#^*P* < 0.05 vs. sex-matched WT mice; ^‡^*P* < 0.05 vs. male group of the same genotype.

At electron microscopic analysis, mild mitochondria damage was evident in both male and female H-AMPKα1 WT mice at 18 h after CLP and was characterized by a few swollen mitochondria (Figure 2). H-AMPKα1 WT mice of both sexes also exhibited an increased number of autophagosomes and autolysosomes with sequestrated materials when compared to basal levels of control mice. There was also a significant increase of elongated mitochondrial morphology in H-AMPKα1 WT mice of both sexes after CLP. On the contrary, male H-AMPKα1 KO mice exhibited a significantly higher structural damage of mitochondria at 18 h after CLP when compared to male WT mice. Structural damage was characterized by the presence of swollen organelles with distorted cristae, translucent matrix and disrupted membrane. Interestingly, mitochondrial damage was significantly lower in KO female mice when compared to WT female mice and KO male mice after CLP; whereas there was no difference in the number of elongated organelles between WT and KO mice of both sexes. H-AMPKα1 WT and KO mice also exhibited an increased number of autophagosomes and autolysosomes with sequestrated materials when compared to basal levels of control mice. However, WT female mice had a higher number of autophagosomes and autolysosomes when compared to WT male mice after sepsis. Interestingly, number of autophagosomes was significantly lower in female KO mice when compared to sex-matched WT mice and to male KO mice after sepsis. To determine whether hepatocyte-specific deficiency of AMPKα1 might affect energy homeostasis, we measured ATP levels in the liver. There was no change in ATP levels after sepsis in male or female WT mice when compared to baseline conditions of WT sex-matched control animals (Figure 2L). However, male KO experienced a significant decrease in ATP levels at 18 h after CLP when compared to baseline conditions of KO control animals. Interestingly, there were no changes in ATP content after sepsis in female KO mice. Thus, hepatocyte-specific AMPKα1 deficiency promotes mitochondrial damage in a sex-dependent manner.

### Hepatocyte-Specific Deficiency of AMPKα1 Influences Liver Autophagy After Sepsis in a Sex-Dependent Manner

**Figure 3:**
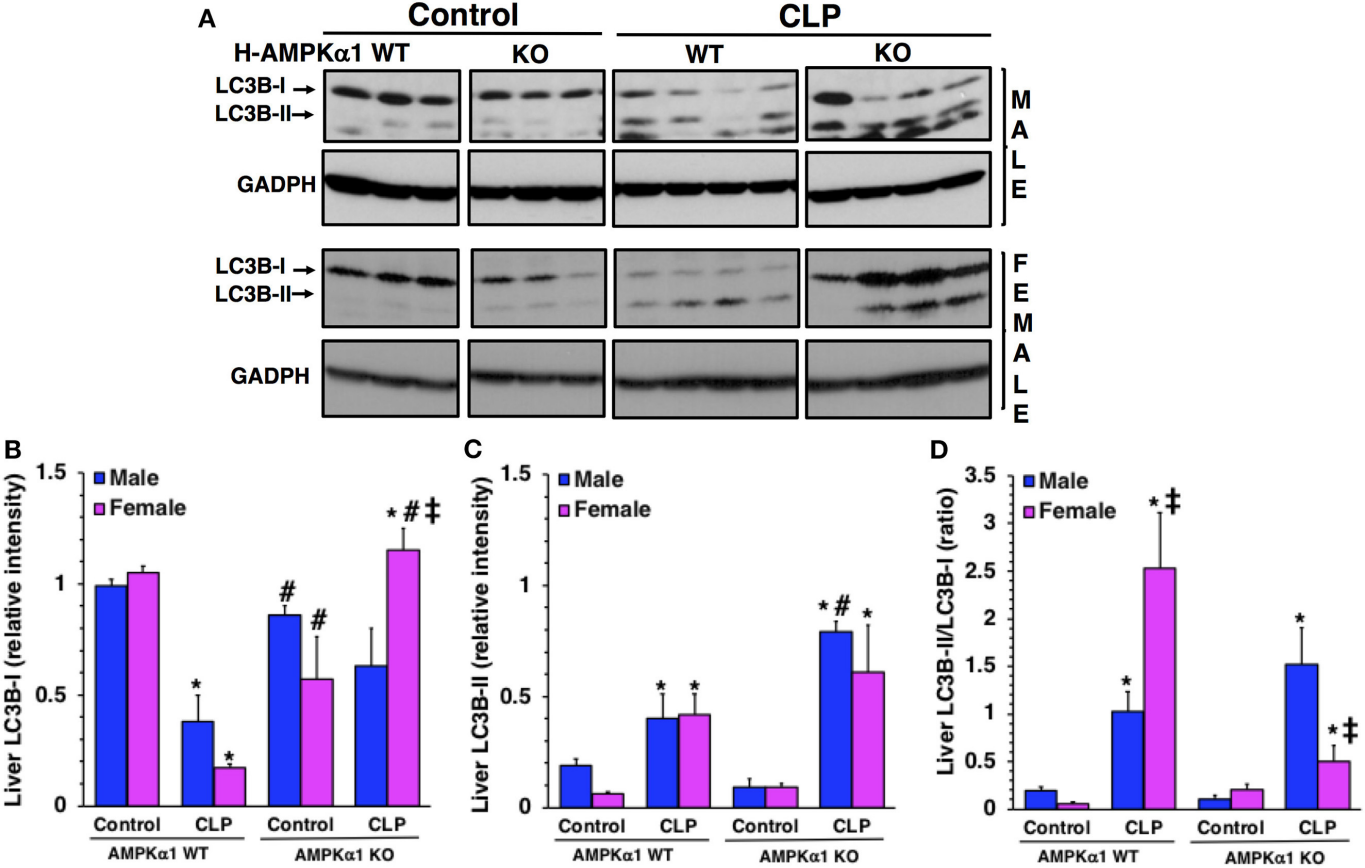
Representative Western blots of protein expression of LC3B-I and LC3B-II in liver cytosol extracts; GADPH was used as loading control protein **(A)**. Image analyses of cytosol relative intensity of LC3B-I **(B)**, LC3 B-II **(C)** and LC3B-II/LC3B-I **(D)** as determined by densitometry. Each data represents the mean ± SEM of 3–4 animals for each group. *P < 0.05 vs. sex-matched control mice; ^#^*P* < 0.05 vs. sex-matched WT mice; ^‡^*P* < 0.05 vs. male group of the same genotype.

To confirm the number of autophagosomes observed at electron microscopy, we further quantified the process of autophagy in the liver by evaluating the expression of the light chain 3B protein (LC3B) (Figure 3). The protein is converted from a cytosolic LC3B-I form to a conjugated LC3B-II form in the autophagosomal membrane and correlates with autophagic vesicle formation (22). At 18 h after CLP, LC3B-II/LC3B-I ratio significantly increased in both male and female WT mice when compared to baseline content of sex-matched control mice. However, WT female mice had a higher ratio when compared to WT male mice after sepsis, further confirming the higher number of autophagosomes seen at electron microscopy. Interestingly, female KO mice exhibited a higher expression of LC3BI when compared to male KO mice after sepsis. Consequently, LC3B-II/LC3B-I ratio was significantly lower in female KO mice when compared to sex-matched WT mice and to male KO mice after sepsis. There was no difference in LC3B-II/LC3B-I ratio between WT and KO mice in the male group. Thus, hepatocyte-specific AMPKα1 deficiency influences autophagy in a sex-dependent manner.

### Hepatocyte-Specific Deficiency of AMPKα1 Results in Lung Injury After Sepsis in a Sex- Dependent Manner

**Figure 4:**
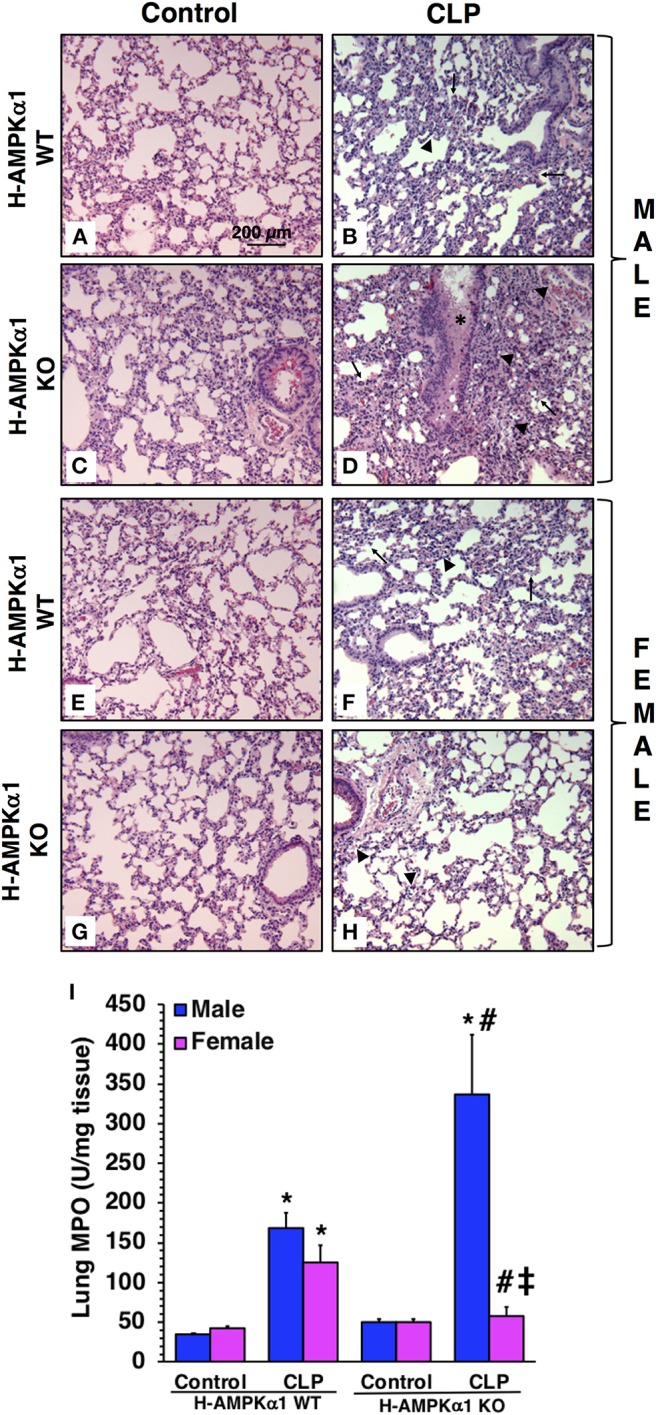
Representative histology photomicrographs of lung sections of hepatocyte-specific (H-AMPKα1) wild-type (WT) and knock-out (KO) male **(A–D)** and female **(E–H)** mice at baseline conditions (Control) or 18 h after cecal ligation and puncture (CLP). Lung damage in H-AMPKα1 WT male **(B)** and female **(F)** mice with modest infiltration of inflammatory cells (*arrow heads*) and reduced alveolar space (*arrows*). Lung damage in H- AMPKα1 KO male mice **(D)** with reduced alveolar space, bronchial congestion (*asterisk*) and accumulation of red and inflammatory cells. Mild lung damage in H-AMPKα1 KO female mice **(H)** with mild infiltration of inflammatory cells only. Magnification ×100; scale bar = 200 μm. A similar pattern was seen in *n* = 4–8 different tissue sections in each experimental group. Lung myeloperoxidase (MPO) activity **(I)** in male and female H- AMPKα1 WT and KO mice at 18 h after CLP. Data represents the mean ± SEM of 4–8 mice for each group. *P < 0.05 vs. sex-matched control mice; ^#^*P* < 0.05 vs. sex-matched WT mice; ^‡^*P* < 0.05 vs. male group of the same genotype.

To obtain insight into the role of hepatocyte AMPKα1 in the development of multiple organ failure we also evaluated lung injury by histology. At 18 h after CLP, both male and female H-AMPKα1 WT mice exhibited similar lung damage with modest infiltration of inflammatory cells and reduced alveolar space (Figure 4). However, male H-AMPKα1 KO mice exhibited marked lung injury characterized by reduced alveolar space, alveolar and bronchial congestion and accumulation of red and inflammatory cells when compared to male WT mice (Figures 4A–D). Male KO mice also exhibited higher lung MPO when compared to WT male mice (Figure 4I). Interestingly, the H-AMPKα1 KO female mice had lower MPO levels than WT female mice after CLP. Also, lung injury was milder (Figures 4E–H) and tissue MPO (Figure 4I) levels were significantly lower in KO female mice when compared to KO male mice after CLP. Thus, hepatocyte-specific AMPKα1 deficiency influenced the inflammatory response in the lung in a sex-dependent manner.

### Hepatocyte-Specific Deficiency of AMPKα1 Influences Systemic Production of Cytokines in a Sex-Dependent Manner

**Figure 5:**
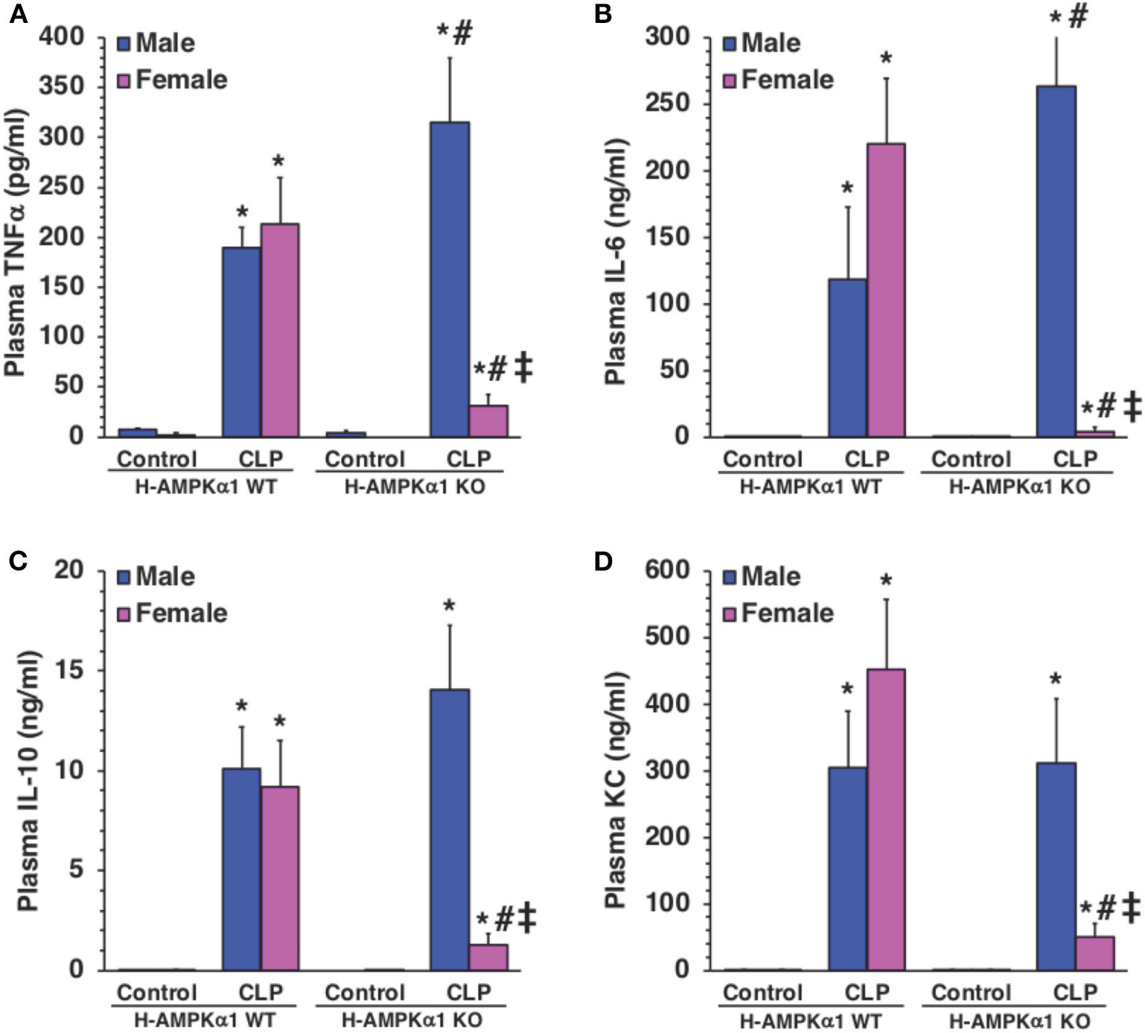
Plasma levels of TNFα **(A)**, IL-6 **(B)**, IL-10 **(C)**, and KC **(D)**. Data represents the mean ± SEM of 4–8 mice for each group. *P < 0.05 vs. sex-matched control mice; ^#^*P* < 0.05 vs. sex-matched WT mice; ^‡^*P* < 0.05 vs. male group of the same genotype.

To determine whether hepatocyte AMPKα1 influenced the systemic production of cytokines during sepsis, plasma levels of TNFα, IL-6, IL-10, and KC were measured. At 18 h after CLP, both male and female WT mice exhibited a significant elevation in plasma levels of all cytokines compared with sex-matched control mice. However, male KO mice had significantly higher levels of TNFα and IL-6 after sepsis when compared to male WT septic mice (Figures 5A,B), while levels of IL-10 and KC were similar in both WT and KO male mice (Figures 5C,D). Interestingly, plasma levels of cytokines were significantly lower in KO female mice when compared to WT female mice and KO male mice after CLP (Figure 5). Thus, hepatocyte-specific AMPKα1 deficiency influenced the systemic inflammatory response in a sex-dependent manner.

### Hepatocyte-Specific Deficiency of AMPKα1 Exacerbates Sepsis-Induced Mortality in Male Mice Only

**Figure 6:**
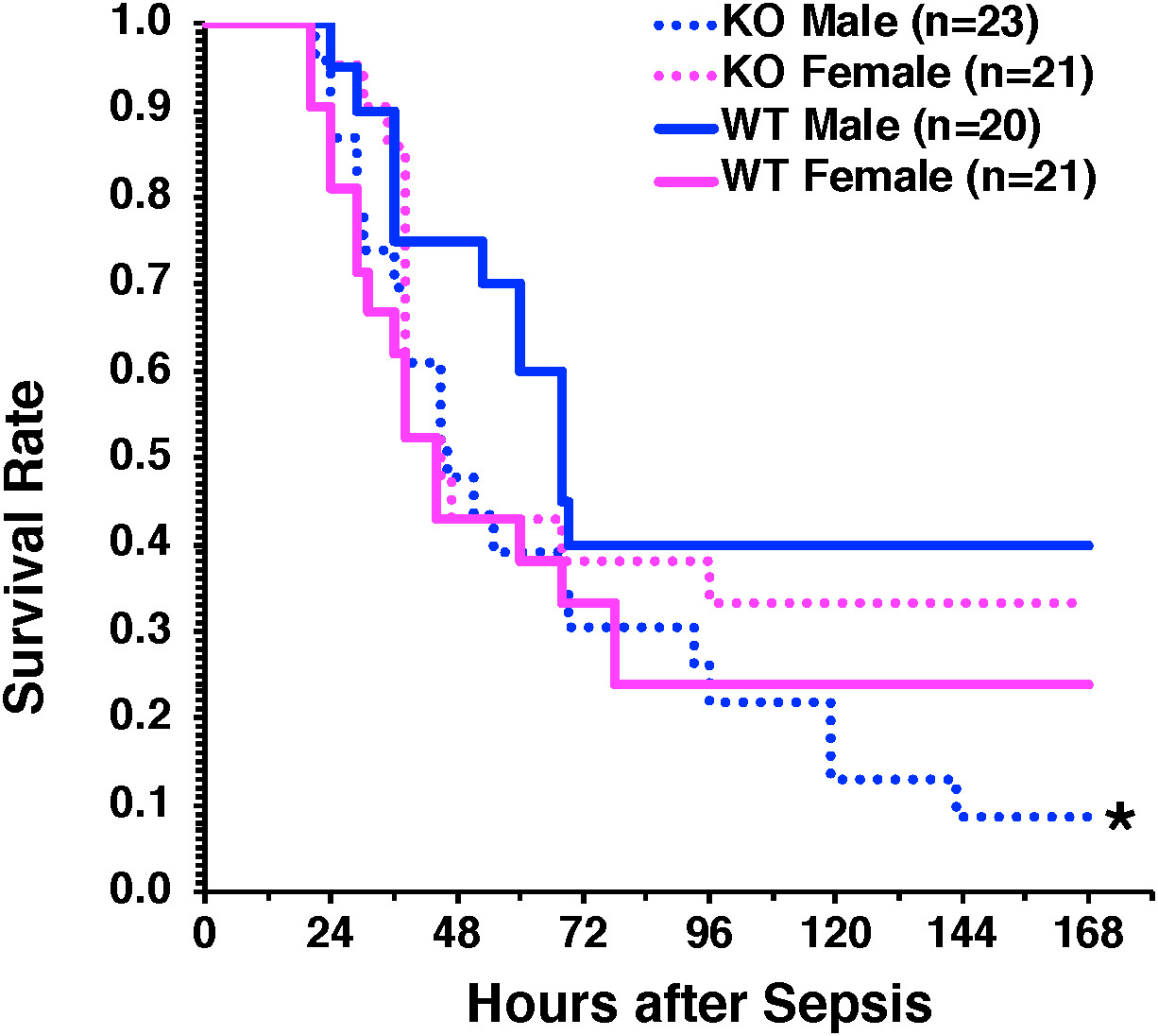
Survival rate of hepatocyte-specific (H- AMPKα1) wild-type (WT) and knock-out (KO) mice at 7 days after cecal ligation and puncture (CLP). Mice were subjected to CLP and received fluid resuscitation (35 ml/kg normal saline with 5% dextrose subcutaneously) immediately after, at 3 h and every 24 h after the CLP procedure up to 72 h. *P < 0.05 of KO males vs. WT males as determined by Gehan-Breslow analysis.

To confirm the protective role of hepatocyte AMPKα1 in long-term outcomes of sepsis, we performed a model of CLP with low mortality at 48 h after CLP (<20% mortality) but delayed high mortality between 3 and 7 days after CLP. Male H-AMPKα1 KO mice experienced higher mortality (91%) than WT mice (60%, *P* < 0.05) at 7 days after CLP. However, this effect was not observed in female H-AMPKα1 KO mice, which had similar mortality rate as female WT mice (67% in KO mice and 76% in WT mice) (Figure 6).

## Discussion

Being responsible of important physiological functions, such as detoxification, energy production, hormonal and immune balance, and coagulation, the liver is a critical organ for host survival following severe injury, including sepsis (6). Among sepsis patients, liver dysfunction or failure is associated with mortality rates ranging from 54 to 68%, higher than the mortality rates of sepsis patients with respiratory system dysfunction or failure (23–25). Despite these data of poor prognosis, the pathophysiology of liver dysfunction and its potential role in influencing multiple organ failure remain unclear. In the present study we have demonstrated that hepatocyte AMPKα1 is a key regulator of liver metabolic and innate immune function. Specifically, we have provided evidence that hepatocyte-specific AMPKα1 deletion worsened liver and lung injury and exacerbated the lethal effects of sepsis in young male mice. The underlying cellular mechanisms of this excessive vulnerability to injury related to increased neutrophil infiltration in liver and lung and increased systemic production of TNFα and IL-6. However, the most intriguing observation made here was that, in young female mice with hepatocyte-specific AMPKα1 deletion, the signs of liver injury did not coincide with worsening of sepsis. On the contrary, hepatocyte-specific AMPKα1 deficiency in female mice was associated with protective effects in the lung and reduced systemic inflammatory response without affecting survival. Thus, our data demonstrate that AMPKα1 modulates the metabolic and inflammatory responses in the liver in a sex-dependent manner.

AMPK is the master regulator of diverse metabolic events to maintain energy homeostasis. The protein consists of a catalytic α-subunit and two regulatory β- and γ-subunits. Two isoforms have been identified of the α-subunit (1 and 2) and β-subunit (1 and 2) and three isoforms of the γ-subunit (1, 2, and 3). Both AMPKα1 and α2-containing complexes are present in the liver in equal distribution in rodent models (11, 12, 18), but AMPKα1 is the predominant form in human hepatocytes (17). In previous studies, we have demonstrated that pharmacological activation of AMPK ameliorated liver injury in young and mature male animals subjected to CLP by regulating autophagy and gene transcription of mitochondrial structural and transport proteins, and metabolic enzymes (14). In this current study, despite sharing some histological and biomarkers similarities of liver injury, male and female H- AMPKα1 KO mice exhibited quite different liver mitochondrial features, as KO female mice did not exhibit ultrastructural damage compared with WT littermates. On the contrary, H- AMPKα1 KO female mice had the least mitochondrial damage and maintained normal ATP production among all groups of septic mice. In regard to this discrepancy between histological injury and changes in mitochondrial damage, it must be noted that such conflicting results have also been reported in clinical studies. Autopsy studies revealed a discordance between histologic findings and the degree of organ dysfunction of patients who died of sepsis (26). Cell death in the heart, kidney, liver, and lung was relatively minor and did not reflect the clinical evidence of more profound organ dysfunction (26). Furthermore, examination of *post mortem* organs from septic patients identified abnormalities of mitochondrial structures in cardiomyocytes, but cardiomyocyte cell death was rarely observed despite the magnitude of organ dysfunction (27). Taken together, our data suggest that maintenance of metabolic stability is associated with resilience to deleterious effects of sepsis and further confirm that mitochondrial damage is the major pathophysiologic characteristic of organ failure as seen in human sepsis.

Clinical studies have reported increased autophagy in multiple organs and tissues in sepsis (28). AMPK induces autophagy by inhibiting the mammalian target of rapamycin pathway (10, 13). Autophagy is a critical step in maintaining mitochondrial quality control by selective removal of damaged organelles (13). There is experimental evidence that disruption of autophagy is associated with organ dysfunction. For example, using a young rat model, Chien et al. demonstrated that liver autophagy occurred early during sepsis, but it declined at later time points, when it was associated with liver dysfunction (29). On the contrary, AMPK-mediated autophagy has been shown to attenuate mitochondrial dysfunction in endotoxin stimulated hepatocytes (30). Mitochondrial morphology is also an adaptive response to cellular metabolic demands and an elongated pattern of mitochondria has been associated to mechanisms of fission/fusion to maximize ATP production (31). However, male H-AMPKα1 WT had less autophagosomes than WT female mice. At examination of molecular events, the conversion of LC3B from its free form (LC3B-I) to its phosphatidyl-ethanol-amine-conjugated form (LC3B-II) is a required step in autophagosome formation. Therefore, the LC3B-II/LC3B-I can be considered as a reliable marker to quantify autophagy (22). In our study, the LC3B-II/LC3B-I ratio was higher after sepsis in the liver of H-AMPKα1 WT female mice, thus suggesting a better capability to mount an autophagic event in response to cellular stress. Interestingly, female KO mice exhibited a reduction of autophagosomes when compared to WT littermates after sepsis. This event was also consistent with lower number of altered mitochondria in female KO mice when compared to male KO mice, thus suggesting that mitochondrial damage is an important requisite for upregulation of autophagy. Therefore, our data further suggest that AMPKα1 influences organ function in a sex-dependent manner also through finely tuning of autophagy according to the degree of mitochondrial impairment.

In concert with Kupffer cells, other immune competent and non-hematopoietic resident cells, hepatocytes produce a complex cytokine milieu of both pro-inflammatory and anti-inflammatory cytokines, which contribute to the initiation and amplification of the systemic acute phase response (32, 33). In our study, we observed that AMPKα1 deficiency in male mice enhanced systemic production of TNFα and IL-6, thus suggesting that AMPKα1 modulates the acute phase response. Consistent with our observation, it has been demonstrated that pharmacological activation of AMPK suppressed immune stimulated inflammation in mouse and human hepatocytes (34). Interestingly, in our study the specific inactivation of AMPKα1 in the liver in male mice led to an exacerbation of lung injury, thus suggesting that liver metabolic disturbance affects distant organ function. Therefore, the results presented here, employing a mouse model for genetic specific inactivation of AMPKα1 in hepatocytes, further extend the evidence for the liver as a key driver of immune and inflammatory responses during sepsis and strongly validate AMPK as a potential therapeutic target.

Paradoxically, specific deficiency of AMPKα1 in hepatocytes in female mice led to a remarkable reduction in the systemic inflammatory response and protected against sepsis-induced lung injury. Because AMPKα1 deficiency was not accompanied with liver metabolic defects in female mice, these sex-dependent differences point to other liver compensatory mechanisms independent by AMPKα1 in young female. In this regard, it is possible that estrogen may exert a compensatory effect for AMPKα1 deletion. For example, previous studies reported that double global genetic deficiency of AMPKα1 and AMPKα2 in female mice did not affect the inflammatory process of osteoarthritis; whereas estrogen prevented articular cartilage destruction (35, 36). Since in our study male and female H-AMPKα1 WT mice exhibited similar organ injury and outcomes after sepsis, we speculate that the differential hormonal milieu between male and female may, indeed, result in a diversification of mitochondria quality control during acute stress only in condition of dysregulation of AMPKα1 and the level of expression *per se* of this enzyme is most likely a key element for sex-dependent switch of metabolic regulation. Further comprehensive studies and elucidations of mitochondrial bioenergetics are required to better understand the signaling pathways and molecules involved in this switch.

## Conclusions and Limitation

Our findings are based on cross-sectional data comparing young age-matched male and female mice, future longitudinal studies are needed to disentangle age and sexual maturation effects, including the impact of ovariectomy and orchiectomy, steroid hormonal levels and the hepatoprotective effects of estrogen during sepsis. We used male and female mice at the age of 8–12 weeks old, which are in their peak reproductive age (37). Therefore, their comparison with older groups of mice is necessary to establish association with levels of estrogens and decline of sexual maturation. Furthermore, a broad investigation of bioenergetic and biosynthetic mitochondrial functionalities by omics technologies should be included to identify novel sex-related mitochondrial pathomechanisms. Nevertheless, by means of cell type-specific gene knockout technology, the current study provides conclusive data that AMPKα1 expression in hepatocytes functions as a protective factor against sepsis in young male mice. However, paradoxically findings of our study point out that sex-dependent differences exist in the susceptibility to sepsis in the context of AMPKα1 deficiency. As AMPK activators, such as metformin, are widely used for diabetes type II in the clinical arena (38), further insight into the exact biological mechanism of AMPK may help to provide better clarity as to the potential pharmacological targeting of AMPK in sepsis.

## Data Availability Statement

The datasets generated for this study are available on request to the corresponding author.

## Ethics Statement

The animal study was reviewed and approved by the Institutional Animal Care and Use Committee of the Cincinnati Children's Hospital Medical Center.

## Author Contributions

SK and BZ conceived and designed the projects. SK and VW performed the animal experiments. SK, GP, PL, and MO'C performed the biochemical assays. SK, GP, and KR performed the electron microscopy analysis. SK, GP, and BZ analyzed the data and prepared graphics. SK and BZ wrote the draft of the manuscript. All authors reviewed and approved the final manuscript.

### Conflict of Interest

The authors declare that the research was conducted in the absence of any commercial or financial relationships that could be construed as a potential conflict of interest.
